# Antibacterial Activity of *Coffea robusta* Leaf Extract against Foodborne Pathogens

**DOI:** 10.4014/jmb.2204.04003

**Published:** 2022-07-08

**Authors:** Atchariya Yosboonruang, Atcharaporn Ontawong, Jadsada Thapmamang, Acharaporn Duangjai

**Affiliations:** 1 Division of Microbiology, School of Medical Sciences, University of Phayao, Phayao 56000, Thailand; 2 Unit of Excellence in Research and Product Development of Coffee, Division of Physiology, School of Medical Sciences, University of Phayao, Phayao 56000, Thailand

**Keywords:** Coffee leaf extract, antibiotics, membrane disruption, membrane potential, foodborne pathogen

## Abstract

The purpose of this study was to examine the phytochemical compounds and antibacterial activity of *Coffea robusta* leaf extract (RLE). The results indicated that chlorogenic acid (CGA) is a major component of RLE. The minimum inhibitory concentrations (MICs) of RLE against *Staphylococcus aureus*, *Bacillus subtilis*, *Escherichia coli*, and *Salmonella* Typhimurium were 6.25, 12.5, 12.5, and 12.5 mg/ml, respectively. RLE effectively damages the bacterial cell membrane integrity, as indicated by the high amounts of proteins and nucleic acids released from the bacteria, and disrupts bacterial cell membrane potential and permeability, as revealed via fluorescence analysis. Cytotoxicity testing showed that RLE is slightly toxic toward HepG2 cells at high concentration but exhibited no toxicity toward Caco2 cells. The results from the present study suggest that RLE has excellent potential applicability as an antimicrobial in the food industry.

## Introduction

Microorganisms that cause food spoilage or contamination are a worldwide problem that leads to substantial food waste. According to Gustavsson *et al*. [1], global food loss has reached 40% due to a variety of reasons including microbial contamination. Microorganisms, notably bacteria, yeasts, and molds, can grow on food and produce metabolites by consuming nutrients, which causes food spoiling [2]. Besides food spoilage, microorganisms can cause foodborne diseases [3, 4] and represent a seriously problem for public health and food safety [5]. *Bacillus cereus*, *Staphylococcus aureus*, *Escherichia coli*, and *Salmonella* spp., the major causes of foodborne bacterial illnesses, are widespread in the environment, including water, air, and dust, and can easily contaminate food products [6]. Several pathogens have developed resistance to antimicrobials, and this has also become a significant worldwide problem [7, 8]. Due to increasing antimicrobial resistance and the adverse effects of some synthetic antimicrobials, the need to develop natural ones that are non-cytotoxic but have high antibacterial efficacy has become paramount [9]. Food preservation that does not affect the taste or nutritional value but ensures food safety and a long shelf-life is essential for the food industry [10]).

The development of natural antimicrobial substances such as food additives for controlling foodborne pathogens has become a hot topic [9, 11], and therefore, many researchers are now interested in discovering and researching new preservative compounds from natural extracts. Those from various medicinal plants have revealed antioxidant and antimicrobial properties due to their active compounds, such as flavonoids, alkaloids, terpenoids, and tannins [12]. Many plant extracts, such as ginger, garlic, basil, and tea exhibit antibacterial activity against both gram-positive and gram-negative bacteria [13, 14].

Coffee is one of the more intriguing plants to be researched as a food additive, and coffee extract has been reported to exhibit antimicrobial activity against both gram-negative and gram-positive bacteria such as *Bacillus* spp., *S. aureus*, *Enterococcus* spp., *E. coli*, and *P. aeruginosa*, among others [15-17]. Duangjai *et al*. [18] revealed the antibacterial activity of coffee pulp extract against gram-positive bacteria (*S. aureus* and *S. epidermidis*) and gram-negative bacteria (*P. aeruginosa* and *E. coli*). Several researchers have reported that the antibacterial activities and antioxidant properties of coffee extract could be due to phenolic acids, tannin, CGA, caffeine, malic acid and other compounds [19, 20]. One possible mechanism may enable these compounds to disrupt cell membrane permeability [19]. Moreover, some researchers believe that coffee melanoidins inhibit bacterial growth via metal chelation [21, 22]. However, the mechanism of action of coffee extracts on bacterial cells is still unclear.

Consequently, in this study, we investigated the phytochemicals and antibacterial activity of *Coffea robusta* leaf extract (RLE), and further, we examined the antibacterial mechanism on bacterial membrane damage concerning the potential disruption in the membrane of microorganisms.

## Materials and Methods

### Strains and Inoculum Preparation

The bacteria used were obtained from the Thailand Institute of Scientific and Technological Research, including both gram-positive bacteria (*S. aureus* (TIRST1840), *B. cereus* (TIRST7), and *B. subtilis* (TIRST1928)), gram-negative bacteria (*P. aeruginosa* (TIRST357), *E. coli* (TIRST527), and *S.* Typhimurium (TIRST1469)). Inoculums were prepared by suspending colonies of the bacteria grown on tryptic soy agar (TSA) and incubating them at 37°C for 24 h before adjusting the turbidity to McFarland standard No. 0.5.

### Chemicals

All of the bacterial culture media were purchased from HiMedia Laboratories (India). Antimicrobial agents were purchased from Oxoid Limited (UK). Dulbecco’s minimum essential medium (DMEM, high glucose), bovine serum albumin, and fetal bovine serum (FBS) were purchased from Invitrogen (USA).

### Coffee Leaf Extract Preparation

*C. arabica* and *C. robusta* leaves were obtained from Chao-Thai-Pukao Factory (Thailand). The coffee leaves were rinsed with distilled water and dried in an oven at 55°C before grinding with a blender. Extract from the ground leaves was obtained by boiling them in water (100 ± 3°C) for 10 min (the coffee leaf powder to hot water ratio was 1:5 w/v and the process was repeated three times). The filtered solution was then lyophilized to yield crude aqueous extracts. *C. arabica* leaf extract (ALE) and *C. robusta* leaf extract (RLE) were kept at -20°C until used.

### Phytochemical Analysis

**Determination of the Total Phenolic Content**

Total phenolic content was measured by using the Folin-Ciocalteu assay [23]. In brief, the reaction was prepared by mixing 20 μl extracts (1 mg/ml) with 100 μl of 10% (w/v) Folin-Ciocalteu reagent followed by adding 80 μl of 1.5% Na_2_CO_3_ solution in a 96-well plate. The mixture was then kept in the dark at room temperature for 30 min, after which absorbance was measured at 750 nm by using a spectrometer (BioTek, USA). Total phenolic content was recorded as mg GAE (gallic acid equivalent)/g of dry extract.

### High-Performance Liquid Chromatography (HPLC) Analysis

This was carried out on an Agilent 1200 series HPLC instrument (Agilent Technologies, USA) and a Zorbax Eclipse XDB-C18 column (4.6 × 150 mm, particle size 5 μm) for 40 min at a flow rate of 0.6 ml/min and an injection volume of 20 μl. Mobile phase A (15% methanol) and mobile phase B (85% methanol: deionized water (30:70)) with the addition of 2% acetic acid (pH 3.4) was used as the eluent. UV detection of polyphenol chlorogenic acid (CGA) at 320 nm and phytochemical caffeine at 280 nm was conducted on the eluate. The amounts of the compounds are expressed as mg/g extract

### Determination of Antibacterial Activity

After exploring the polyphenols and phytochemicals of ALE and RLE, RLE was chosen for further study. The antibacterial properties of RLE were investigated by using the agar well diffusion method. Briefly, TSA plates were prepared by pouring 20 ml of TSA onto plates and allowing it to solidify. Bacterial inoculums (10^7^ CFU/ml) were swabbed onto the TSA before being punched with a 6 mm sterile Cork borer. The extract was applied to each well at a concentration of 200 mg/ml. Gentamicin and sterile distilled water were used as positive and negative controls, respectively. The plates were incubated at 37°C for 18–24 h before measuring the diameters (mm) of the inhibition zones. To assess the reproducibility of the results, the experiments were carried out three times.

### Determination of the Minimum Inhibitory Concentration (MIC)

The MICs of the extracts were determined by using the Clinical and Laboratory Standards Institutés [24] broth microdilution method. Two-fold serial dilutions of the samples using 0.85% NaCl (final concentration ranging from 1.56 -200 mg/ml) were prepared and added to sterile 96-well plates. The bacterial strains were added to a final concentration of 5 × 10^5^ CFU/ml to individual wells, after which the plates were incubated at 37°C for 24 h. MIC is defined as the minimum concentration of RLE that inhibited bacterial growth.

### Kinetic Assay

Time-kill kinetic assay for RLE at 1x, 2x, and 4x MIC was conducted against *S. aureus*, *B. subtilis*, *E. coli*, and *S.* Typhimurium using the previous method [25] with few modifications. The bacteria were incubated with each RLE concentration and the growth of bacteria was investigated for 24 h (0, 1, 2, 4, 8, 16, 24 h). Bacterial growth without RLE was used as the control. At each time interval, the suspension of the sample was individually spread on a TSA plate. After incubation, the colonies were counted.

### Effect of RLE on Bacterial Cell Membrane Integrity

The leakage of proteins and nucleic acids is a measure for investigating the cell membrane integrity of bacteria treated with RLE. To measure the leakage of proteins, the tested bacteria were washed and re-suspended in phosphate-buffered saline (PBS), and then the turbidity was adjusted to obtain 1 × 10^8^ CFU/ml. The bacterial suspensions were incubated with RLE at concentrations of 0, 1, 1.5, and 2 MIC for 1 h. After filtration through a 0.22 µm filter membrane, the supernatants were collected and diluted with PBS. The leakage proteins were investigated by using the DC assay kit (Bio-Rad Laboratories Ltd., USA) with fluorescence detection at 750 nm using a Cytation 5 Multi-mode Microplate Reader (BioTek), while the protein concentration was calculated by comparing with a standard protein. Meanwhile, absorbance at 260 nm was used to measure the leakage of nucleic acids by using a NanoDrop Lite spectrophotometer (Thermo Scientific, USA).

### Effect of RLE on Cell Membrane Potential

To investigate the membrane potential, the tested bacteria were treated with various concentrations of RLE by following the previous method with some modifications [26, 27]. Bacteria were grown in TSB for 18 h before being collected by centrifugation at 2,500 *×*g for 10 min. The pellets were washed twice before being re-suspended in NaCl solution. Bacterial suspensions (1 × 10^8^ CFU/ml) were separated and treated for 1 h with RLE at concentrations of 0, 1, 1.5, and 2 MIC. The RLE-treated suspension (1 ml) was centrifuged, washed, and re-suspended in 0.5% NaCl. Afterward, 200 ml of bacterial suspension was mixed with Rhodamine 123 in a 96-well plate to a final concentration of 10 mg/ml. The samples were then incubated in the dark at 37°C for 10 min before being washed and re-suspended in 0.5% NaCl. Finally, the fluorescence intensity was measured using a fluorescence spectrophotometer (BioTek) at excitation and emission wavelengths of 480 and 530 nm, respectively.

### Effect of RLE on 1-N-Phenylnapthylamine (NPN) Uptake

The method to determine NPN uptake was performed with some modifications to the previous method [6, 28]. After 1 h of treatment with RLE (0, 1, 1.5, or 2 MIC) as described above, cells were collected, rinsed, and re-suspended in 0.5% NaCl solution. Then, 200 μl aliquots of the bacterial suspension were added to wells on a 96-well plate and then mixed with 100 mM NPN to finally obtain 0.75 mM, after which the fluorescence intensity was immediately measured by using a fluorescence spectrophotometer (BioTek). NPN has an excitation wavelength of 350 nm and an emission wavelength of 420 nm.

### Effect of RLE on Cell Viability

The cytotoxicity of RLE against HepG2 and Caco2 cells was assessed by using the MTT assay modified by Xu *et al*. [6]. HepG2 or Caco2 cells were cultured at 37°C in 5% CO_2_ in a cell culture medium (DMEM containing 10%FBS and 1% penicillin/streptomycin). Then, 200 μl aliquots of cells at a density of 1 × 10^4^ cell/well were plated on 96-well plates and incubated for 24 h at 37°C in a humidified, 5% CO_2_ environment. The extracts were added to the wells at various concentrations (100, 200, 400, 800, or 1,000 g/ml) and incubated for 24 h at 37°C in 5% CO_2_. PBS solution was used as a control. After that, MTT solution (5 mg/ml) was added to the test plate, which was then incubated for 4 h at 37°C. Formazan crystals were dissolved in dimethyl sulfoxide and the fluorescence was measured at 490 nm by using a microplate reader (BioTek).

### Statistical Analysis

All experiments were carried out in triplicate and the results are reported as the mean ± SD. One-way analysis of variance (ANOVA) and Dunnett’s test were used to analyze the data via R4.1.1 and significance was set as *p* < 0.05.

## Results

### Total Phenolic Content and Phytochemical Compounds in the Coffee Leaf Extracts

**Fig. 1:**
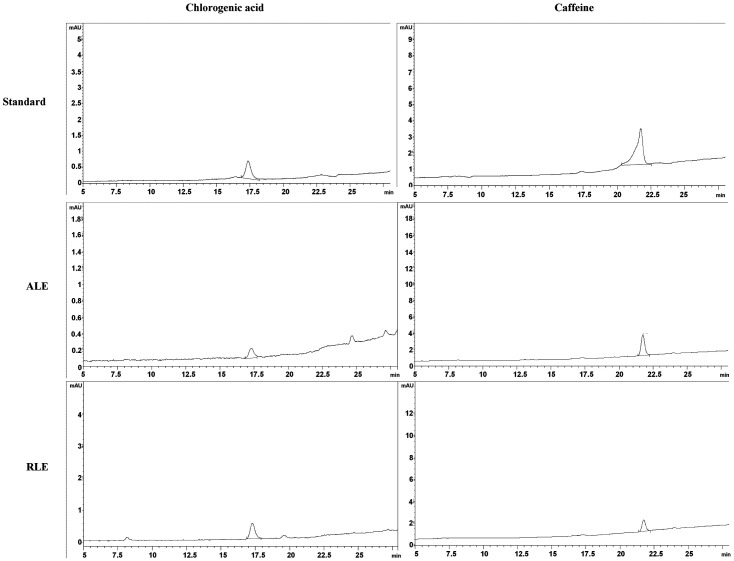
HPLC Chromatogram of *Coffea arabica* leaf extract (ALE) and *Coffea robusta* leaf extract (RLE) at 320 nm for chlorogenic acid and 280 nm for caffeine.

The total phenolic compound amounts in ALE and RLE were 324.37 ± 13.70 and 357.59 ± 26.50 mg GAE/100 g, respectively. Meanwhile, the amounts of CGA and caffeine in ALE were 5.15 and 14.2 mg/g extract, respectively, while in RLE they were 33.5 and 14.1 mg/g extract, respectively (Fig. 1).

### Antibacterial Activity

The antibacterial activity test results showed that RLE inhibited all six tested bacteria, with inhibition zones for *S. aureus*, *B. cereus*, *B. subtilis*, *P. aeruginosa* *E. coli*, and *S.* Typhimurium of 9.94 ± 0.17, 18.67 ± 0.07, 9.96 ± 0.22, 9.92 ± 0.15, 9.98 ± 0.23, and 6.78 ± 0.43 mm, respectively (Table 1). The MIC values of RLE for *S. aureus*, *B. cereus*, *B. subtilis*, *P. aeruginosa* *E. coli* and *S.* Typhimurium were 6.25, 50, 12.5, 25, 12.5, and 12.5 mg/ml, respectively. According to the MIC of RLE investigations, bacteria showing MIC ≤ 12.5 mg/ml (against *S. aureus*, *B. subtilis*, *E. coli*, and *S.* Typhimurium) were selected for a further mechanistic study, the data for which are reported in Table 1.

### Bacterial Time-Kill Kinetic Assay

**Fig. 2:**
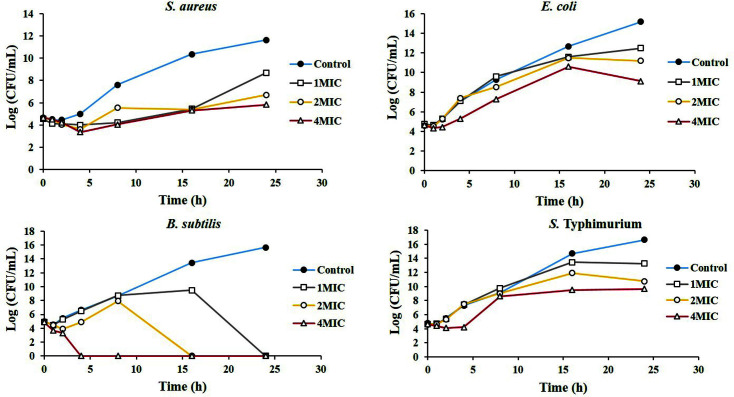
Time-kill kinetic assay of *Coffea robusta* leaf extract (RLE) at a concentration of 1-4 MIC against *S. aureus*, *B. subtilis*, *E. coli*, and *S.* Typhimurium. The MIC of *S. aureus* was 6.25 mg/ml and the MIC of *B. Subtilis*, *E. coli*, and *S.* Typhimurium were 12.5 mg/ml.

Bactericidal activity of RLE was investigated using 1- to 4-fold MIC by time-kill kinetic assay. Time–kill curve analysis was presented in Fig. 2. The result revealed that RLE had bactericidal activity against *B. subtilis* within1-4 h. RLE only showed bacteriostatic activity against other bacteria when increasing the concentration up to 4x MIC.

### Effect of RLE on Cell Membrane Integrity

**Fig. 3:**
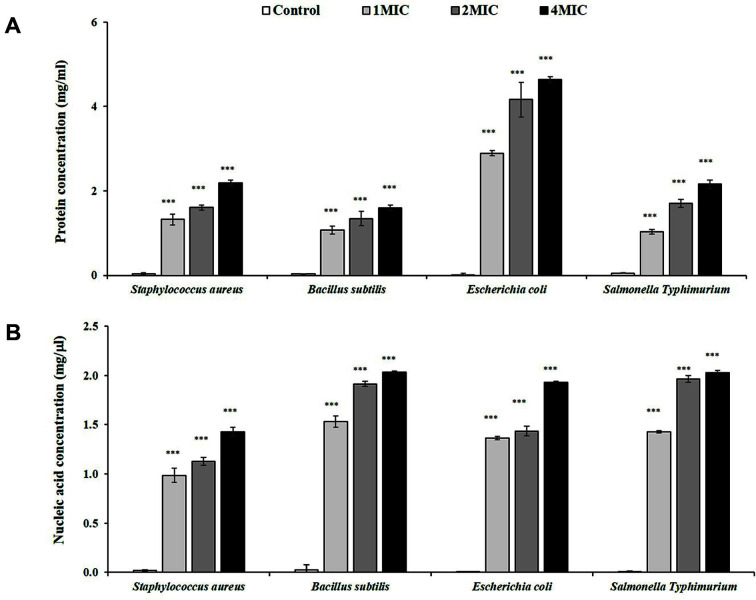
Release of proteins (**A**) and nucleic acids (**B**) from tested bacteria treated with *Coffea robusta* leaf extract (RLE). The plotted values are the mean and the bars are the standard deviation (*n* = 3). ***, *p* < 0.001 compared to the control group. MIC, minimum inhibitory concentration. The MIC of *S. aureus* was 6.25 mg/ml and the MIC of *B. Subtilis*, *E. coli*, and *S.* Typhimurium were 12.5 mg/ml.

The findings from this study demonstrate that the leakage of proteins and nucleic acids of bacterial cells was dose-dependent and gradually increased when the concentration of RLE (1, 1.5, and 2 MIC) increased (1.32, 1.60, 2.19 mg/ml, and 2.89, 4.16, 4.63 mg/ml, respectively), as shown in Figs. 3A and 3B. Particularly high protein leakage was observed in both gram-positive (*S. aureus*) and gram-negative (*E. coli*) bacteria. For the nucleic acid leakage, *B. subtilis* and *S.* Typhimurium exhibited a high nucleic acid concentration when treated with 1.0, 1.5, and 2.0 MIC of RLE (1.531, 1.912, 2.034 mg/ml and 1.426, 1.965, 2.025 mg/ml, respectively).

### Effect of RLE on Membrane Potential

**Fig. 4:**
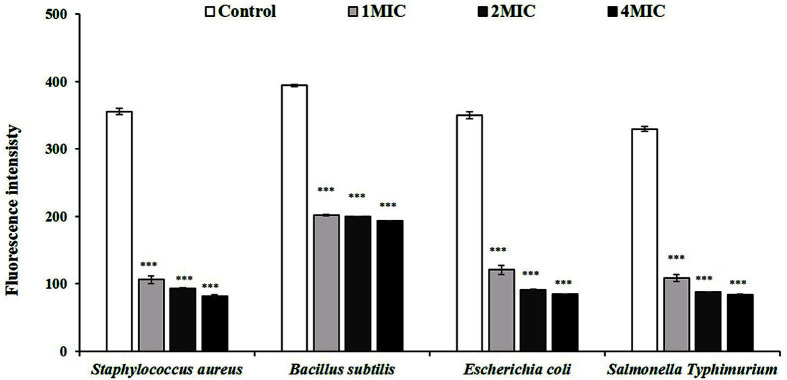
Membrane potential disruption was measured as the reduced Rhodamine 123 fluorescence intensity of the tested bacteria treated with *Coffea robusta* leaf extract (RLE). The plotted values are the mean and the bars are the standard deviation (*n* = 3). ***, *p* < 0.001 compared to the control group. MIC, minimum inhibitory concentration. The MIC of *S. aureus* was 6.25 mg/ml and the MIC of *B. Subtilis*, *E. coli*, and *S.* Typhimurium were 12.5 mg/ml.

As shown in Fig. 4, the fluorescence intensity of bacteria suspensions treated with RLE was significantly lower than the control. The fluorescence levels for *S. aureus* treated with RLE at 1.0, 1.5, or 2.0 MIC decreased by 70.18%, 73.93%, and 77.02%; for *B. Subtilis* by 48.87%, 49.43%, and 50.96%; for *E. coli* by 65.54%, 75.85%, and 73.39%; and for *S.* Typhimurium by 73.92%, 67.07%, and 74.57%, respectively. Thus, when the concentration was increased, the fluorescence intensity was slightly reduced.

### Effect of RLE on NPN Uptake

**Fig. 5:**
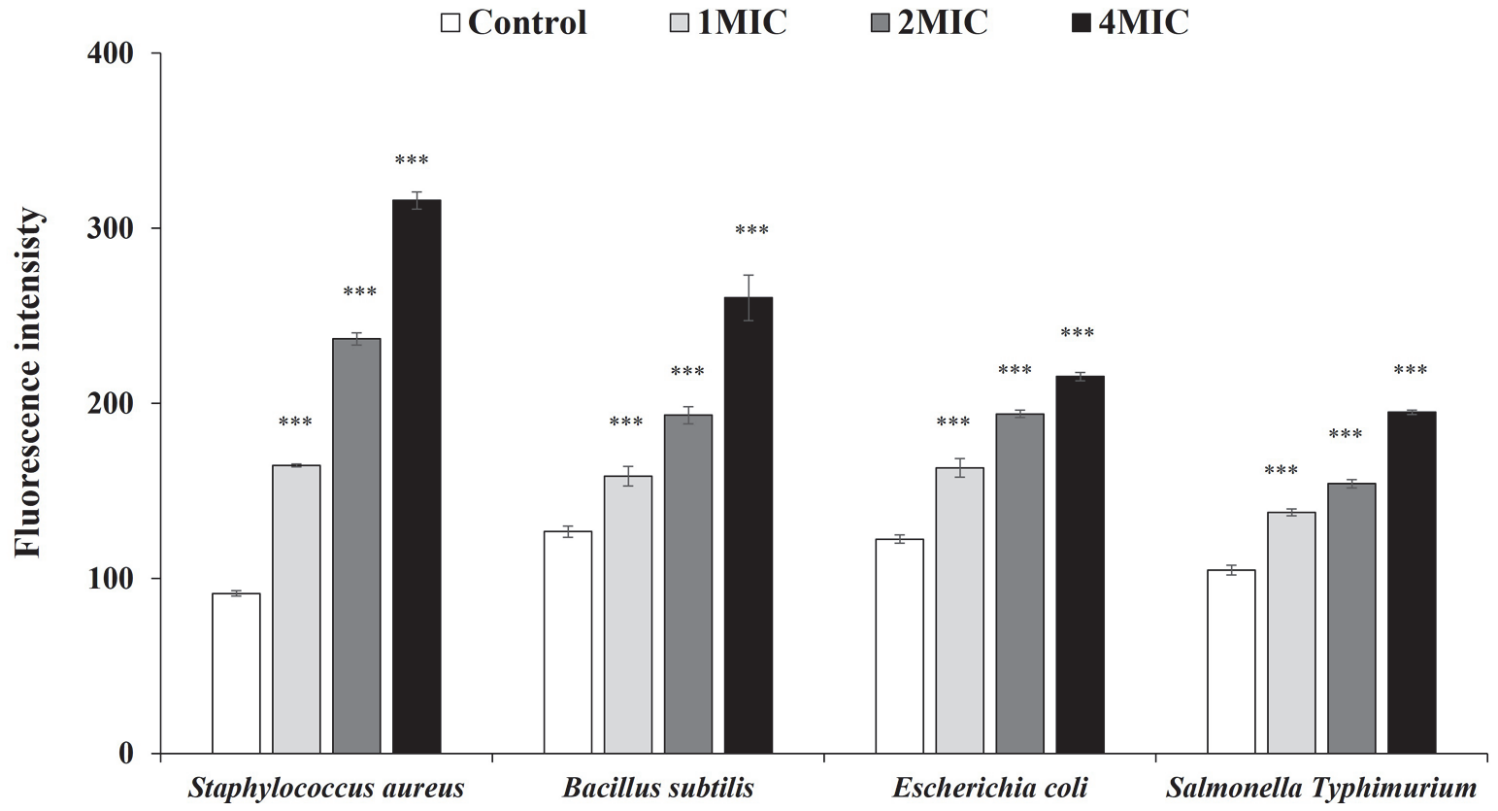
Fluorescence intensity due to NPN uptake by the tested bacteria treated with *Coffea robusta* leaf extract (RLE). The plotted values are the mean and the bars are the standard deviation (*n* = 3). ***, *p* < 0.001 compared to the control group. MIC, minimum inhibitory concentration. The MIC of *S. aureus* was 6.25 mg/ml and the MIC of *B. Subtilis*, *E. coli*, and *S.* Typhimurium were 12.5 mg/ml.

As shown in Fig. 5, a higher NPN uptake was observed in all tested bacteria after treatment with RLE compared with the control. Of the gram-positive bacteria, the fluorescence intensity of *S. aureus* was higher than that of *B. subtilis*, while of the gram-negative bacteria, the fluorescence intensity of *E. coli* was higher than that for *S.* Typhimurium. Moreover, the increase in NPN uptake was in a concentration-dependent manner. Compared with the control group at 1.0, 1.5, and 2.0 MIC of RLE, *S. aureus* increased NPN uptake by 79.99%, 159.0%, and 245.34%; *B. subtilis* by 24.97%, 52.39%, and 105.34%; *E. coli* by 33.27%, 58.35%, and 75.74; and *S.* Typhimurium by 31.47%, 47.01%, and 85.89%, respectively.

### Effect of RLE on Cell Viability

**Fig. 6:**
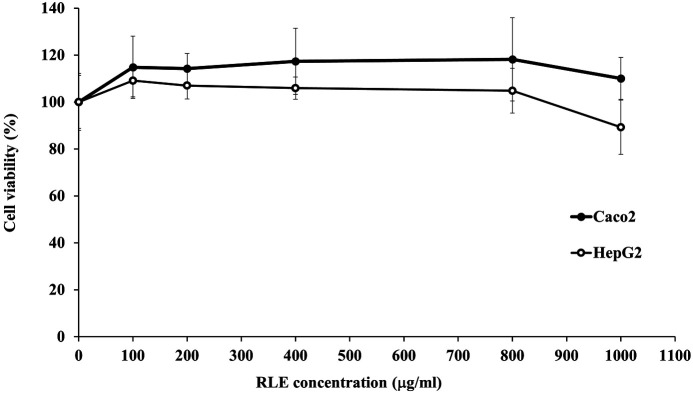
The effect of *Coffea robusta* leaf extract (RLE) concentration on HepG2 and Caco2 cell viability. The plotted values are the mean and the bars are the standard deviation (*n* = 3).

The result showed that RLE was non-toxic to HepG2 at low concentration and slightly toxic at high concentration while no cytotoxic effect on Caco2 cells was observed (Fig. 6).

## Discussion

Although the phytochemical and biological activities of coffee extracts have been extensively studied worldwide, this is not the case for coffee leaf extract. In particular, scant information is available on the antibacterial activity and mechanism of RLE. *C. arabica* pulp and leaf extracts contain a wide range of phytochemicals, including phenolic acids, flavonoids, terpenoids, alkaloids, catechins, and tannins [29, 30]. Especially, we investigated phenolic acid (CGA) and alkaloid caffeine and found that both ALE and RLE contained similar caffeine content whereas the CGA content in RLE was 6.5 times higher than in ALE. Hence, the high CGA content in RLE piqued our interest and was further studied. There are several reports on the health benefits of these two compounds, particularly CGA, which has revealed antioxidant, anti-inflammatory, antimicrobial, antidiabetic, and neuroprotective properties [31]. Stauder *et al*. [22] discovered that the high-molecular-weight components in coffee extract have anti-adhesive and anti-biofilm properties against *Streptococcus mutans*; however, there have been few reports on the antibacterial mechanisms against foodborne pathogens.

The results of the current study indicate that RLE can inhibit both gram-positive and gram-negative foodborne pathogens as efficaciously as coffee bean extract can. Moreover, we explored its possible mechanism of action and applicability as a natural antimicrobial food additive. Hence, our findings can serve as the foundation for future research into the bacteriostatic properties of coffee leaf extract. The antibacterial activity of RLE was directly determined via MIC assays. The RLE was serially diluted 2-fold to obtain concentrations of 0.156, 3.125, 6.25, 12.5, 25, 50, 100, or 200 mg/ml. We found that RLE has antibacterial activity against the tested bacteria in the MIC range of 6.25 to 50 mg/ml. RLE had bactericidal activity against *B. subtilis* while it was bacteriostatic against *S. aureus*, *E. coli*, and S. Typhipurium. The sample used in this study was only the crude extract. The efficacy of the killing activity was not that high compared with the pure component. However, previous studies showed time-kill kinetic assay results for CGA, caffeic acid, and caffeine (the main components of RLE) against pathogens, including multidrug-resistant *Vibrio cholerae* [25, 32, 33] Thus, the antibacterial activity of RLE could be due to the high CGA and some caffeine concentration with support from other compounds in the extract.

After bacteria were treated with RLE, the leakage of both nucleic acids and proteins was observed, implying damage to the cell membrane integrity of *S. aureus*, *B. subtilis*, *E. coli*, and *S.* Typhimurium had occurred. These results correspond with those from previous studies on various natural extracts [6, 9]. These studies also noted that intracellular material leakage inhibits protein and DNA synthesis, which kills the bacteria. The bacterial cell membrane plays an important role in preserving the shape of the organism to allow the permeation of biochemicals, and synthesizing ATP. When the cell membrane is disrupted, cell cytoplasm, nucleic acids, proteins, and several granules can leak into the surrounding environment, thereby causing the bacterium to die.

According to Xu *et al*. [6], bacteria treated with Lachnum YM30 intracellular melanin had lower fluorescence intensity due to Rhodamine 123 than the control group, which is consistent with the results of the present study; we found that when bacteria were treated with RLE, the intensity of their fluorescence due to Rhodamine 123 decreased significantly compared to the control group, which indicates that the cell membranes of the bacteria had been damaged [26, 27]. Membrane potential, which is the difference in electric potential between the inside and outside of the cell, is referred to as membrane potential and can be used to determine whether or not the cell membrane has been damaged. Rhodamine 123, a lipophilic and cationic dye, can be used to evaluate membrane potential [27].

NPN, a nonpolar probe, has been widely used to monitor biological membrane permeability. It exhibits strong fluorescence in the presence of phospholipids in solution but weak fluorescence in aqueous solution [28]. Normally, a biological membrane has the ability to extrude external hydrophobic molecules, which prevents NPN from being taken up by the cell. When the membrane is disrupted or dysfunctional, NPN can be taken up, which causes the fluorescence intensity to increase. In this study, both gram-positive and gram-negative bacteria showed gradually increased emission intensity as the RLE concentration was increased. However, unlike the previous finding that gram-positive bacteria had higher initial fluorescence than gram-negative bacteria [6, 34], our results found them to be similar.

Because of the potential applicability of RLE as a food additive or food preservative, HepG2 and Caco-2 cells were used to study its cytotoxicity. We found that RLE was non-toxic to HepG2 cells at a low dose and only slightly toxic at the high dose while no evidence of cytotoxicity was prevalent in Caco-2 cells. These results correlate with those from other studies [6, 35], in which no relationship between the antimicrobial effects and cytotoxicity of the extract was discovered. Thus, the concentration required to kill bacteria is lower than the concentration that produces cytotoxicity to mammalian cells.

We extracted the contents of *C. robusta* leaves and determined that RLE shows bactericidal activity against foodborne pathogens without cytotoxic effects to mammalian cells. This is the first report on the antibacterial effect and mechanisms of RLE on both gram-positive and gram-negative bacteria. Thus, RLE shows remarkable potential as a naturally antimicrobial substance with wide application in the food industry.

## Figures and Tables

**Table 1:** Antibacterial activity of RLE against the tested bacteria compared with the positive control (gentamycin) and the negative control (distill water).

Tested bacteria	Inhibition Zone (mm)			MIC (mg/ml)

	RLE	+ control	- control	
*S. aureus*	10.22 ± 0.02	27.2 ± 0.00	-	6.25
*B. cereus*	19.52 ± 0.07	27.25 ± 0.00	-	50
*B. subtilis*	11.23 ± 0.01	29.15 ± 0.00	-	12.5
*P. aeruginosa*	9.96 ± 0.12	26.88 ± 0.02	-	25
*E. coli*	10.44 ± 0.24	27.1 ± 0.00	-	12.5
*S.* Typhimurium	10.11 ± 0.16	26.85 ± 0.02	-	12.5

*remark; RLE, Coffea robusta leaf extract; MIC, minimum inhibitory concentration.
